# Bio-Based Bisbenzoxazines with Flame Retardant Linker

**DOI:** 10.3390/polym13244330

**Published:** 2021-12-10

**Authors:** Thorben Sören Haubold, Laura Puchot, Antoine Adjaoud, Pierre Verge, Katharina Koschek

**Affiliations:** 1 Fraunhofer Institute for Manufacturing Technology and Advanced Materials IFAM, Wiener Strasse 12, 28359 Bremen, Germany; Thorben.haubold@ifam.fraunhofer.de; 2 Department 2 Biology/Chemistry, University of Bremen, Leobener Straße 7, 28359 Bremen, Germany; 3 Materials Research and Technology Department, Luxembourg Institute of Science and Technology, 5 Avenue des Hauts-Fourneaux, L-4362 Esch-sur-Alzette, Luxembourg; laura.puchot@list.lu (L.P.); Antoine.adjaoud@list.lu (A.A.); pierre.verge@list.lu (P.V.); 4 Department of Physics and Materials Science, University of Luxembourg, 2, Avenue de l’Université, L-4365 Esch-sur-Alzette, Luxembourg

**Keywords:** flame retardancy, benzoxazine, halogen-free, bio-based

## Abstract

This work explores the strategy of incorporating a highly substituted reactive flame retardant into a benzoxazine moiety. For this purpose, a DOPO-based flame retardant received a chain extension via reaction with ethylene carbonate. It was then reacted with phloretic acid to obtain a diphenol end-capped molecule, and further reacted with furfurylamine and paraformaldehyde to obtain a benzoxazine monomer via a Mannich-like ring closure reaction. This four-step synthesis yielded a partly bio-based halogen-free flame retardant benzoxazine monomer (DOPO-PA-fa). The successful synthesis was proven via NMR, IR and MS analysis. The polymerization behavior was monitored by DSC and rheological analysis both showing the polymerization starts at 200 °C to yield pDOPO-PA-fa. pDOPO-PA-fa has a significant thermal stability with a residual mass of 30% at 800 °C under ambient atmosphere. Furthermore, it reached a V-0 rating against small flames and an *OI* of 35%. Blended with other benzoxazines, it significantly improves their thermal stability and fire resistance. It emphasizes its potential as flame retardant agent.

## 1. Introduction

Polybenzoxazines are thermosetting materials with excellent properties, such as a near-zero shrinkage upon polymerization [1], high mechanical and thermal properties [2]. They have gained a lot of attention during the last decade since many different bio-sourced synthons can be used to design their molecular structure, which simply require a primary amine, a phenol non-substituted in *ortho* position and an aldehyde. For instance, furfurylamine [3], stearylamine [4], chitosan [5] and amines, based on rosin [6], isomannide [7] and vanilin [8] have been employed as amines; additionally, guaiacol [4,9], sesamol [10,11], cardanol [7,12,13], eugenol [14] or phloretic acid (PA) [15,16] have been used as naturally occurring phenolic compounds. The design of formaldehyde-free monomers is now hyping, with the recent works of Caillol et al. [17,18], and Ishida and Froimowicz et al. [11].

Even if benzoxazines exhibit a naturally good intrinsic flame retardancy, the demand to increase their fire properties is significant enough to have initiated many research studies. The flame retardancy (FR) of polymers can be improved by different approaches: the use of unreactive [19] or reactive additives [20], and the incorporation of flame retardant groups into the monomer structure [21]. Among the flame retardant groups, 9,10-dihydro-9-oxa-10-phosphaphenanthrene-10-oxide (DOPO) is a widely investigated halogen-free reactive additive that is mainly gas phase active yielding PO and HPO radicals upon decomposition. It has the advantage to trap energetic radicals and to improve the reaction against small flames [22]. DOPO is an efficient FR as additive or chemically incorporated in a molecular structure, e.g., epoxies [23,24] or polyester [25,26,27]. DOPO derivatives, e.g., dopotriamine [21] or dopotriol [28], were incorporated into benzoxazine to improve its flame retardancy. Partly bio-based bisbenzoxazines were described by combining DOPO with bio-based bisphenols and the following reaction with furfurylamine and paraformaldehyde [29]. Furthermore, DOPO can be incorporated into the oxazine ring at the 4-position during the ring closing reaction. For this purpose, DOPO reacts with phenol benzaldehyde and different amine functionalities, e.g., tolyl-, *p*-hydroxyphenyl-, *p*-carboxyphenyl- [30], aniline- [31] or diamines-based functionalities [32]. The high aromaticity and the resulting highly ordered structures due to pi-stacking lead to high melting temperatures. This results in a narrow temperature range between melting and polymerization temperature, which impedes the polymerization process of these monomers.

**Scheme 1:**
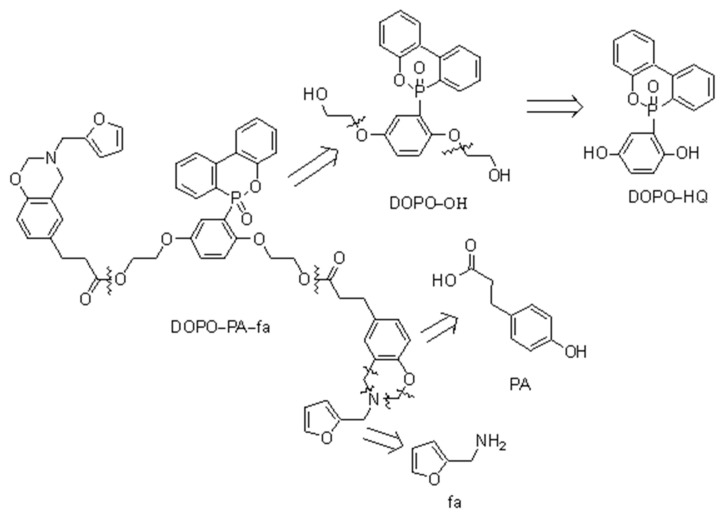
Building blocks for the synthesis of DOPO-PA-fa.

This contribution describes the synthesis and testing of a new bio-based benzoxazine, which is easy to polymerize and has high flame retardancy, in regards to tests against small flames. This was accomplished by the functionalization of DOPO with a phenolic spacer, further functionalized into a benzoxazine. The DOPO linker aims at improving flame retardancy without increasing the melting temperature of the resulting bisbenzoxazine. The linker synthesis was started from the reaction between DOPO and benzoquinone, leading to DOPO-HQ, and followed by a chain extension by reacting DOPO-HQ with ethylene carbonate leading to DOPO-OH, as previously reported by Wang et al. (Scheme 1) [26]. DOPO-OH was thus reacted with phloretic acid to yield DOPO-PA, following a procedure previously reported [15,33,34]. The resulting bisphenolic DOPO linker was finally reacted with furfurylamine and paraformaldehyde to yield the benzoxazine monomer DOPO-PA-fa. The contribution reports on the synthetic steps to achieve the DOPO functionalized bio-based bisbenzoxazine. The novel monomer was then investigated by thermo-analytical methods and rheology in order to describe the polymerization behavior. The thermo-dynamic mechanical stability and reaction against small flames of the resulting polybenzoxazines is also reported in this contribution.

## 2. Materials and Methods

### 2.1. Materials and Reagents

2-Ethoxyethanol, *p*-benzoquinone, dimethylacetamide, ethylene carbonate, 2-propanol, phloretic acid, *p*-toluene sulfonic acid monohydrate, paraformaldehyde, furfurylamine, DOPO, potassium iodide, chloroform and ethyl acetate were supplied from Sigma–Aldrich (Taufkirchen, Germany) and DOPO from TCI (Zwijndrecht, Belgium). All chemicals were used without further purification.

### 2.2. Synthesis

#### 2.2.1. Synthesis of 2-(6-Oxido-6H-dibenz(c,e)(1,2)oxaphosphorin-6-yl)-1,4-hydroxy Phenylene (DOPO-HQ)

The synthesis of DOPO-HQ was adapted by Wang et al. [27]. DOPO (5.40 g, 25 mmol, 1 eq.) was dissolved in 2-ethoxyethanol (20 mL) at 90 °C. After complete solvation, benzoquinone (2.45 g, 22.5 mmol, 0.9 eq.) was slowly portion wise added as a powder, and the reaction mixture was heated to 125 °C for 4 h. After cooling to room temperature, the mixture was filtered, and the powder was recrystallized from 2-ethoxyethanol. The product was dried under reduced pressure at 90 °C for 12 h to obtain an off-white powder in a 73% yield.

^1^H NMR (DMSO-d_6_, 600 MHz, 298 K): δ (ppm) = (assignment, multiplicity, {attribution}, experimental integration, theoretical integration). δ = 9.46 (Ar-OH*, s, {a}, exp 1.00H, th 1.00H); 9.17 (Ar-OH*, s, {b}, exp 1.00H, th 1.00H); 8.27–8.20 (Ar-H*, m, {8,15}, exp 2.08H, th 2.00H); 7.74 (Ar-H*, t, {9}, exp 1.03H, th 1.00H); 7.55–7.62 (Ar-H*, dd, {11}, exp 1.08H, th 1.00H); 7.50 (Ar-H*, td, {10}, exp 1.07H, th 1.00H); 7.45 (Ar-H*, t, {17}, exp 1.04H, th 1.00H); 7.32–7.26 (Ar-H*, m, {16,18}, exp 2.06H, th 2.00H); 7.19 (Ar-CH*, dd, {2}, exp 1.02H, th 1.00H); 6.89 (Ar-H*, dd, {4}, exp 1.03H, th 1.00H); 6.64 (Ar-H*, dd, {5}, exp 1.03H, th 1.00H) ppm.

^31^P NMR (243 MHz, DMSO-d_6_, 298 K) δ = 22.11 (s) ppm.

#### 2.2.2. 2-(6-Oxido-6H-dibenz(c,e)(1,2)oxaphosphorin-6-yl)-1,4-hydroxyethoxy Phenylene (DOPO-OH)

The synthesis of DOPO-OH was adapted by Wang et al. [27]. Dimethylacetamide (100 mL) was poured in a two-neck flask with reflux condenser and nitrogen inlet. Ethylene carbonate (24.44 g, 277.5 mmol, 3 eq.), DOPO-HQ (30.00 g, 92.5 mmol, 1 eq.) and potassium iodide (394 mg, 2.4 mmol, 0.03 eq.) were added. The mixture was heated and stirred at 160 °C for 7 h. After that time, the crude product was precipitated by pouring the solution into water (600 mL). A formed gum was stirred with spatula. After 3 h the gum turned into a solid precipitate which was filtered. The crude product was recrystallized from 2-propanol, filtered and dried at 90 °C under reduced pressure for 14 h. DOPO-OH was obtained as off-white powder in a yield of 62%.

^1^H NMR (DMSO-d_6_, 600 MHz, 298 K): δ (ppm) = (assignment, multiplicity, {attribution}, experimental integration, theoretical integration). δ = 8.29–8.21 (Ar-H*, m, {8,15}, exp 2.4H, th 2.00H); 7.76 (Ar-H*, t, {9}, exp 0.56H, th 1.00H); 7.71–7.65 (Ar-H*, dd, {11}, exp 0.81H, th 1.00H); 7.52 (Ar-H*, td, {10}, exp 1.48H, th 1.00H); 7.46 (Ar-H*, t, {17}, exp 1.17H, th 1.00H); 7.36 (Ar-H*, dd, {16}, exp 0.94H, th 1.00H); 7.33–7.29 (Ar-CH*, m, {18,2}, exp 1.66H, th 2.00H); 7.20 (Ar-H*, dd, {4}, exp 0.81H, th 1.00H); 7.03 (Ar-H*, dd, {5}, exp 1.00H, th 1.00H); 4.87 (CH_2_-OH*, t, {a}, exp 0.85H, th 1.00H); 4.46 (CH_2_-OH*, t, {b}, exp 0.86H, th 1.00H); 4.03–3.91 (Ar-O-CH_2_*, m, {19}, exp 1.39H, th 2.00H); 3.72–3.67 (CH_2_*, m, {20,21}, exp 2.56H, th 3.00H), 3.59–3.52 (Ar-O-CH_2_*, m, {21}, exp 0.82H, th 1.00H); 2.98–2.93 (HO-CH_2_*, m, {19}, exp 1.49H, th 2.00H) ppm.

^31^P NMR (243 MHz, DMSO-d_6_, 298 K) δ = 22.11 (s) ppm.

#### 2.2.3. DOPO-PA

The solventless method was adapted from previous reports [29]. Phloretic acid (26.51 g, 159.5 mmol, 2.2 eq.) was melted in a round-bottom flask at 130 °C. On complete melting, DOPO-OH (29.90 g, 72,5 mmol, 1 eq.) was added portion wise before *p*-toluene sulfonic acid monohydrate (0.70 g, 3.6 mmol, 0.05 eq.) was added. The melt was stirred for 20 h. The glassy product was recrystallized from ethyl acetate to obtain a white powder in a 67% yield.

^1^H NMR (DMSO-d_6_, 600 MHz, 298 K): δ (ppm) = (assignment, multiplicity, {attribution}, experimental integration, theoretical integration). δ = 9.16 (Ar_2_-OH*, t, {a, b}, exp 1.86H, th 2.00H); 8.21–8.13 (Ar-H*, m, {8,15}, exp 2.02H, th 2.00H); 7.74–7.69 (Ar-H*, t, {9}, exp 0.56H, th 1.00H); 7.58–7.50 (Ar-H*, m, {11,10}, exp 1.92H, th 2.00H); 7.45 (Ar-H*, t, {17}, exp 1.07H, th 1.00H); 7.39 (Ar-H*, t, {16}, exp 1.01H, th 1.00H); 7.28–7.21 (Ar-CH*, m, {2,4,18}, exp 1.66H, th 2.00H); 7.03–6.96 (Ar-H*, m, {5,35,40}, exp 2.97H, th 3.00H); 6.94–6.91 (Ar-H*, dt, {25,29}, exp 2.07H, th 2.00H); 6.67–6.63 (Ar-H*, dt, {26,28,37,39}, exp 3.83H, th 4.00H); 4.35 (CH_2_-CH_2_*, t, {20}, exp 1.88H, th 2.00H); 4.25–4.16 (CH_2_-CH_2_*, m, {19}, exp 1.73H, th 2.00H); 3.87–3.80 (Ar-O-CH_2_*, m, {30}, exp 1.01H, th 1.00H); 3.71–3.66 (Ar-O-CH_2_*, m, {30}, exp 0.97H, th 1.00H); 3.49–3.42 (Ar-O-CH_2_*, m, {31}, exp 0.99H, th 1.00H); 3.39–3.35 (Ar-O-CH_2_*, m, {31}, exp 0.48H, th 1.00H); 2.75 (O=C-CH_2_-CH_2_*, t, {23}, exp 1.86H, th 2.00H); 2.59 (O=C--CH_2_*, t, {22}, exp 1.94H, th 2.00H); 2.55–2.51 (O=C-CH_2_-CH_2_**, m, {34}, exp 1.50H, th 2.00H); 2.24–2.02 (O=C- CH_2_**, m, {33}, exp 1.59H, th 2.00H) ppm

^31^P NMR (243 MHz, DMSO-d_6_, 298 K) δ = 21.70 (s) ppm.

MS (ESI) *m/z*, [M+Na]^+^ calculated for C_40_H_37_NaO_10_P^+^, 731.20165; found, 731.20080.

#### 2.2.4. DOPO-PA-fa

Furfurylamine (5.74 g, 59.1 mmol, 2.05 eq.) was mixed with paraformaldehyde (2.04 g, 67.8 mmol, 2.35 eq.) in chloroform (70 mL) and heated to 65 °C for 1 h before the addition of DOPO-PA (20.43 g, 28.83 mmol, 1 eq.), paraformaldehyde (2.04 g, 67.8 mmol, 2.35 eq.) and chloroform (100 mL). The reaction mixture was heated at 65 °C for 48 h. The reaction solution was added to a separation funnel and the organic layer was washed twice with aqueous hydrochloric acid (0.5 M), deionized water, saturated sodium carbonate and brine. The organic layer was pre-dried with magnesium sulfate and filtered, before the chloroform was removed under reduced pressure and the oil was further dried for 14 h at 50 °C. The product was obtained as yellow oil in a yield of 71%.

^1^H NMR (DMSO-d_6_, 600 MHz, 298 K): δ (ppm) = (assignment, multiplicity, {attribution}, experimental integration, theoretical integration). δ = 8.21–8.14 (Ar-H*, m, {8,15}, exp 2.02H, th 2.00H); 7.71 (Ar-H*, t, {9}, exp 0.56H, th 1.00H); 7.60 (fu-CH*, dd, {36,53}, exp 1.37H, th 2.00H); 7.56–7.50 (Ar-H*, m, {11,10}, exp 1.73H, th 2.00H); 7.47–7.42 (Ar-H*, m, {17}, exp 0.97H, th 1.00H); 7.38 (Ar-CH*, t, {16}, exp 1.00H, th 1.00H); 7.28–7.21 (Ar-CH*, m, {2,4,18}, exp 2.93H, th 3.00H); 7.04–6.87 (Ar-H*, m, {5,25,43}, exp 1.84H, th 3.00H); 6.87 (Ar-H*,s, {40,47}, exp 1.13, th 1.00H); 6.76 (Ar-H*,s, {29}, exp 0.74, th 1.00H); 6.68–6.62 (Ar-H*, dt, {44,26}, exp 2.12. H, th 2.00H); 6.44–6.38 (fu-CH*, m, {35,52}, exp 1.63H, th 2.00H); 6.32–6.25 (fu-CH *, m, {34,51}, exp 1.45H, th 2.00H); 4.77 (O-CH_2_*-N, d, {31,49}, exp 2.75H, th 4.00H); 4.41–4.31 (CH_2_-CH_2_*, m, {20}, exp 1.84H, th 2.00H); 4.26–4.16 (CH_2_-CH_2_*, m, {19}, exp 1.23H, th 2.00H); 3.88–3.82 (CH_2_-CH_2_*, N-CH_2_*-Ar, t, {30,37,48}, exp 3.45H, th 5.00H); 3.80 (N-CH_2_*, c, {32,50}, exp 2.91H, th 4.00H); 3.87–3.80 (Ar-O-CH_2_*, m, {37}, exp 1.01H, th 1.00H); 3.71–3.66 (Ar-O-CH_2_*, m, {37}, exp 1.08H, th 1.00H); 3.50–3.44 (Ar-O-CH_2_*, m, {38}, exp 0.70H, th 1.00H); 3.39–3.35 (Ar-O-CH_2_*, m, {38}, exp 0.27H, th 1.00H); 2.75 (O=C-CH_2_-CH_2_*, t, {23}, exp 1.95H, th 2.00H); 2.62 (O=C--CH_2_*, t, {22}, exp 1.55H, th 2.00H); 2.55–2.51 (O=C-CH_2_-CH_2_**, m, {41}, exp 1.20H, th 2.00H); 2.24–2.02 (O=C- CH_2_**, m, {40}, exp 2.13H, th 2.00H) ppm.

^31^P NMR (243 MHz, DMSO-d_6_, 298 K) δ = 19.80 (s) ppm.

MS (ESI) *m/z*, [M+H]^+^ calculated for C_54_H_53_N_2_O_12_P^+^, 951.32524; found, 951,32428.

### 2.3. Equipment and Characterization

An Equinox 55 ATR spectrometer (Bruker, Bremen, Germany) was used to conduct the Fourier transform infrared spectra in attenuated total reflection (ATR) mode using a diamond crystal. The sample were recorded with 32 scans and a resolution of 4 cm^−1^ in a range between 500–4000 cm^−1^.

^1^H-NMR spectra were recorded on an AVANCE NEO 600 MHz-Spectrometer (Bruker, Bremen, Germany) with DMSO-d_6_ as solvent at 20 °C. ^13^C NMR and ^31^P NMR spectra were recorded using the same spectrometer with a frequency of 150 MHz and 243 MHz, respectively. The chemical shifts were reported in parts per million (ppm) and referred to residual protons in the DMSO-d_6_ at 2.50 ppm.

DSC measurements were performed using a Discovery DSC (TA Instruments, Hüllhorst, Germany) with 10 K/min in a range from 0 °C to 300 °C on a specimen scale of 1–2 mg under N_2_ atmosphere. All samples were measured 3 times and the average was taken for evaluation.

DMA experiments were performed using a DMA Q800 (TA Instruments, Hüllhorst, Germany). Thermo-mechanical properties were determined in a temperature range from 0 °C to 250 °C with a heating rate of 2 K/min with a frequency of 1 Hz in a single cantilever bending mode and test specimen of 40 × 10 × 4 mm^3^. *T*_g_ was determined by the maximum of tan(δ).

TGA measurements were carried out with a Q5000 (TA Instruments, Hüllhorst, Germany) with a temperature range from 25 °C to 800 °C and a heating rate of 10 K/min under ambient and nitrogen atmosphere; 2, 5, 20 and 40 K/min were additional heating rates for samples under nitrogen atmosphere.

UL94 vertical burning tests were performed according to DIN EN 60695-11-10 with specimen dimensions of 125 × 13 × 4 mm^3^ and ground edges.

LOI experiments were performed according to EN ISO 4589-2 with specimen type 1, dimensions of 100 × 10 × 4 mm and ground edges with an LOI apparatus (Fire Testing Technology, East Grinstead, UK).

Mass spectrometry (MS) analysis was conducted on a Bruker Impact II (Bruker, Bremen, Germany) mass spectrometer using electrospray ionization with 3 µL/min in DCM/Methanol 1/10 *v/v*.

Rheological measurements were recorded using an Anton Paar Physica MCR 302 rheometer equipped with a CTD 450 temperature control device. Small quantities of the samples were loaded in a parallel plate–plate geometry (ϕ = 25 mm). The polymerization measurements were recorded in the oscillation mode at a frequency of 1 Hz and a controlled strain of 0.1%. Heating ramps of 2 K/min were applied to reach a temperature of 225 °C. The sample deformation was ramped linearly from 1% to 0.2% to remain within the instrument’s limitation and to maintain a linear viscoelastic behavior as the moduli (G′ storage modulus, G″ loss modulus) increase by several orders of magnitude upon curing. The gap between the plates was maintained at 0.5 mm during all the experiments.

The curing was performed with different parameters for the benzoxazines. DOPO-PA-fa was heated up to 105 °C, degassed and poured into a metal mold with the dimension 170 × 130 × 4 mm^3^. The melt was degassed for a second time before polymerizing with the following program 2 h at 170 °C and 2 h at 180 °C. BF-a was heated to 140 °C and p PEG400-PA-fa to 100 °C before transfer to the metal mold. After curing and demolding the specimens were cut according to DMA, LOI and UL94 test dimensions.

## 3. Results

The following paragraphs describe the synthesis and analysis of the bisbenzoxazine DOPO-PA-fa (Scheme 1). The direct use of DOPO-HQ for the benzoxazine synthesis is not possible due to the high degree of substitution and the resulting steric hindrance. Furthermore, the reduced reactivity of its hydroxyl groups is a challenge in the esterification step with PA. Therefore, the chain extension with ethylene carbonate was performed to promote the esterification with PA leading to DOPO-PA.

**Scheme 2:**
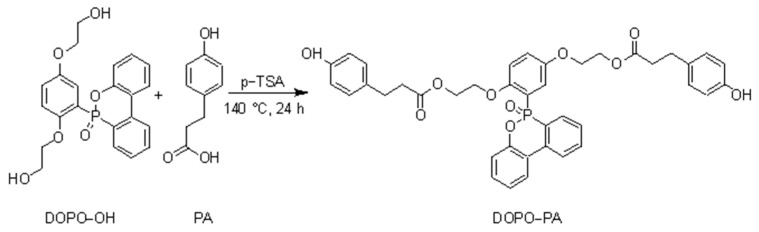
Reaction scheme for the synthesis of the benzoxazine precursor DOPO-PA.

**Figure 1:**
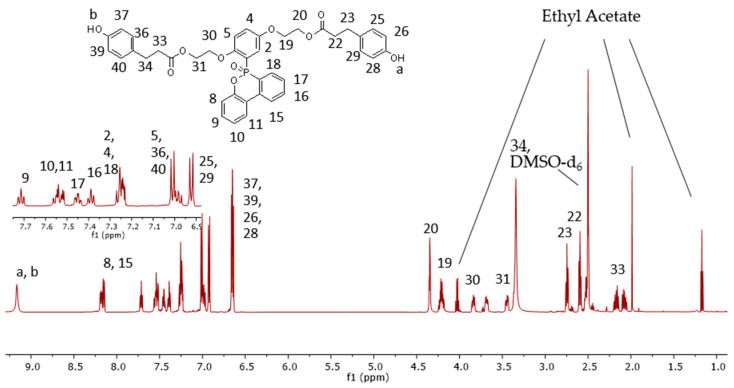
^1^H NMR spectrum of DOPO-PA (in DMSO-d_6_).

The esterification reaction of DOPO-OH and PA was performed in the presence of *p*-TSA (Scheme 2). The structural features of DOPO-PA were determined by ^1^H NMR, FTIR and MS. ^1^H NMR spectrum of DOPO-PA proved the full conversion of DOPO-OH as attested by the disappearance of the aliphatic hydroxyl groups at 4.46 ppm and 4.86 ppm (Figure 1). The signal at 9.17 ppm was assigned to the phenolic hydroxyl group. Furthermore, signals at 4.21 ppm and 4.35 ppm corresponding to a CH_2_-O-CO binding motif confirmed the ester formation.

The different chemical shifts for methylene protons 19 and 20 in comparison with 30 and 31 originated from the asymmetric substitution of the aromatic ring as previously reported by Zhang et al. [27] and Pospiech et al. [25,35]. Accordingly, it can be assumed that this effect is responsible for the different chemical shifts in case of the ester *α*-methylene protons 22 and 33 and the *β* protons 23 and 34. This different chemical shift is reported for polyesters containing DOPO-OH with different aliphatic or aromatic acids showing a regioregularity effect that can only result from non-equal ester *α*-methylene protons [25,35].

Additionally, the sterical effect of the bulky asymmetric DOPO group leads to magnetically deviating protons at position 30, resulting in a further splitting of the signal. As the planar DOPO group is asymmetric and the phenyl rings prevent free rotation, intra- and intermolecular interactions between the ester bonds and the double bond P=O (out of plane) induce a spatial conformation.

**Figure 2:**
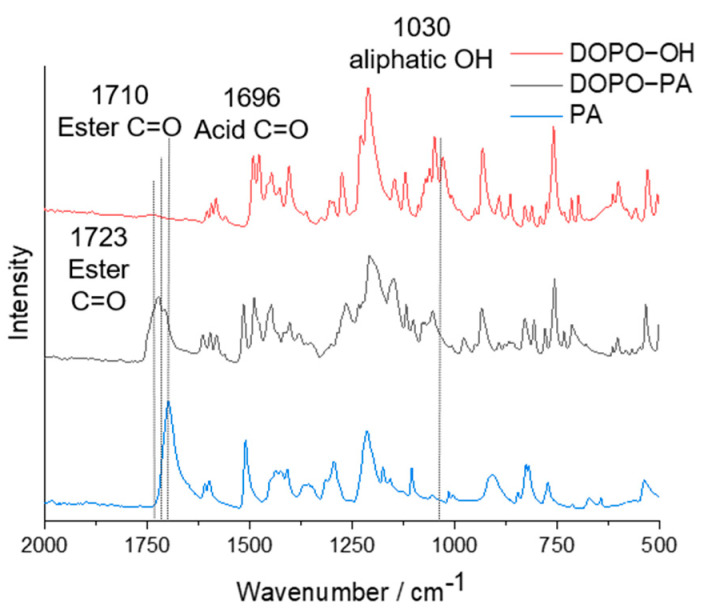
FTIR spectra of DOPO-OH, DOPO-PA and PA.

The ester formation was confirmed by FTIR (Figure 2). A shift of the characteristic peak of the carboxyl group from 1696 cm^−1^ to 1710 cm^−1^ and 1723 cm^−1^ was observed proving the formation of ester linkages. It is further confirmed by the disappearance of the C-O-H stretching signal of DOPO-OH at 1030 cm^−1^, indicating the full consumption of the aliphatic hydroxyl groups.

### 3.1. Synthesis and Characterization of Benzoxazine Monomer Containing DOPO-PA (DOPO-PA-fa)

**Scheme 3:**
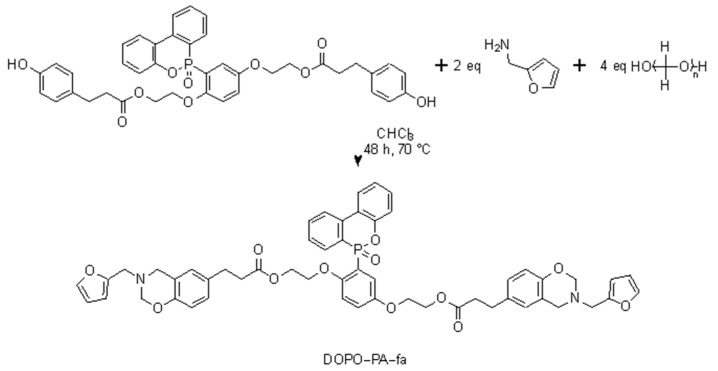
General reaction scheme for the synthesis of DOPO-PA-fa.

DOPO-PA was reacted in chloroform with furfurylamine and paraformaldehyde to yield DOPO-PA-fa (Scheme 3).

**Figure 3:**
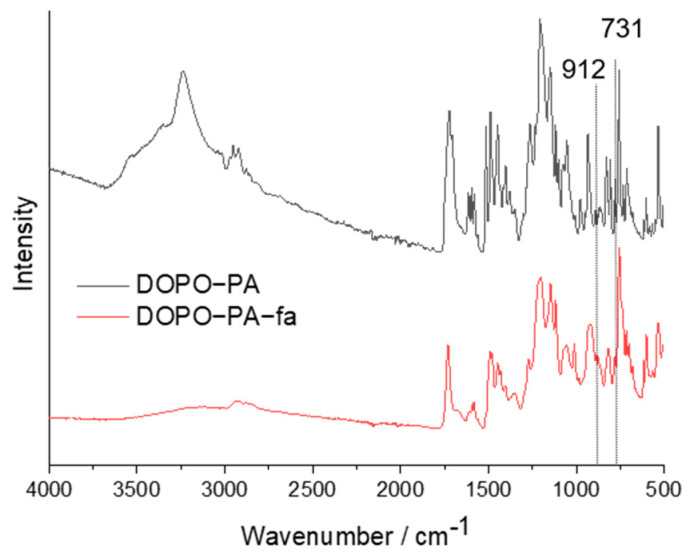
FTIR spectra of DOPO-PA and DOPO-PA-fa.

The benzoxazine ring formation was observed by FTIR (Figure 3). The signal at 912 cm^−1^ was assigned to the ring vibration of benzoxazine rings. The signal at 731 cm^−1^ corresponds to a mono substituted furan ring. Furthermore, the O-H stretching band at 3333 cm^−1^ was reduced significantly, but it was still present, presumably due to an incomplete benzoxazine formation.

**Figure 4:**
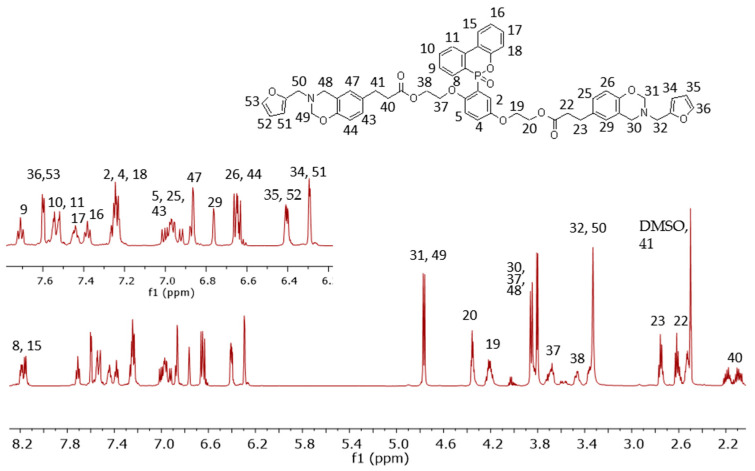
^1^H NMR spectrum in (DMSO-d_6_) of DOPO-PA-fa.

The benzoxazine ring formation was also confirmed by ^1^H NMR (Figure 4). The signals at 4.76 ppm and 3.84 ppm correspond to the protons of O-CH_2_-N and Ar-CH_2_-O, respectively. Furthermore, the protons of the furan ring are identified by the signals at 6.86, 6.41 and 6.29 ppm and the protons of the furfurylamine methylene linkage at 3.80 ppm. As in the case of double bond in DOPO-PA, the P=O bond as well as the lone pair of the tertiary amine (also out of plane) can induce a preferential spatial conformation. Therefore, each type of end-chain oxazine ring methylene peak (30 and 48 or 31 and 49) deviate in the chemical shift, as they are not equivalent. A small residual phenolic hydroxyl group signal at 9.17 ppm indicates that the benzoxazine formation was incomplete, in line with FTIR characterizations. The signal of the O-CH_2_-N and the aromatic signal at 7.71 ppm were set in relation to each other to determine the degree of benzoxazine ring formation. The values of the integration were 2.6 and 1.00, indicating that 65% of the benzoxazine rings are closed.

### 3.2. Thermal Analysis of DOPO-PA-fa

**Figure 5:**
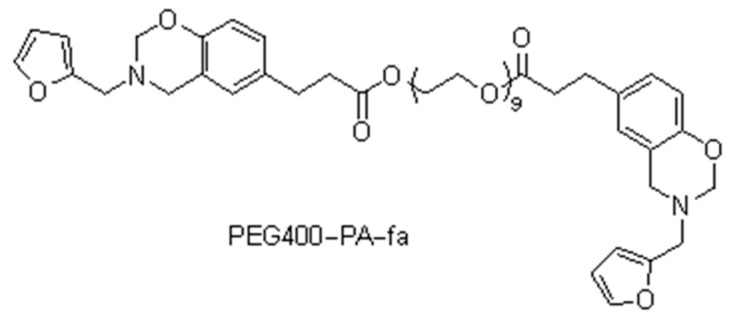
Chemical structure of the reference PEG400-PA-fa [14].

The polymerization of the novel bio-based DOPO-PA-fa was determined by DSC analysis and compared with a conventional bisphenol F and aniline based benzoxazine monomer (BF-a) and a bisbenzoxazine, based on a flexible PEG400 linker (PEG400-PA-fa), as described earlier (Figure 5) [14]. The molecular mass for the spacer group of DOPO-PA-fa and PEG400-PA-fa is similar with approx. 420 g/mol. It is worth mentioning that this reference was selected due to the similar molecular weight of the spacer and their synthesis approach. As the linker of the studied molecule and the reference solely differ in the DOPO functionality, it was expected that the structure of the polymers from DOPO-PA-fa and PEG400-PA-fa would help to identify the contribution of the DOPO moiety.

**Figure 6:**
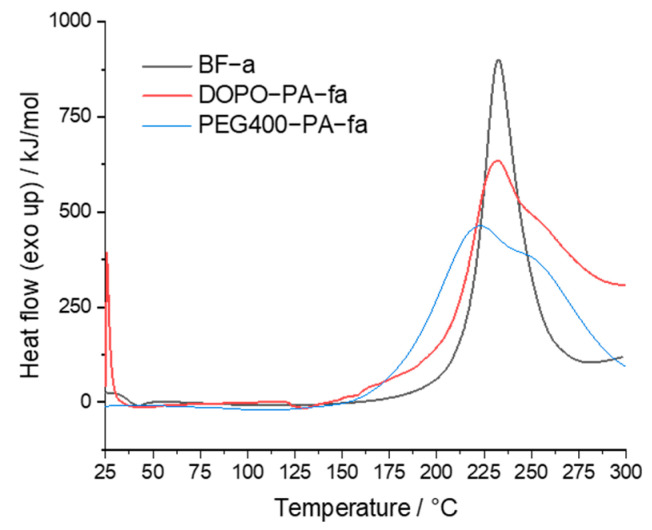
DSC thermograms of BF-a, DOPO-PA-fa and PEG400-PA-fa.

The DSC analysis of DOPO-PA-fa reveals an exothermic signal in the range of 180–260 °C, assigned to the benzoxazine ring-opening polymerization (Figure 6 and Table 1). The temperature onset (*T*_onset_) of DOPO-PA-fa at 208 °C is similar to the *T*_onset_ of BF-a (216 °C) but higher to the *T*_onset_ of PEG400-PA-fa (177 °C). The *T*_onset_ of DOPO-PA-fa is in the range of previously reported benzoxazine containing DOPO [23,27]. The lower *T*_onset_ may, presumably, be due to the residual phenolic impurities observed by FTIR and NMR.

The polymerization enthalpies of BF-a and DOPO-PA-fa are in the same range: 112 and 127 kJ/mol, respectively (Table 1, columns 4 and 5). However, their polymerization profiles differ by the presence of a shoulder for DOPO-PA-fa in the range of 240–250 °C, which is expected to be due to the involvement of the fa group during the polymerization process as shown in previous publications [5,30].

A potential degradation influencing the polymerization enthalpy was excluded as the initial degradation of DOPO-PA-fa at 5% weight loss was occurring at 214 °C (Figure S5).

**Figure 7:**
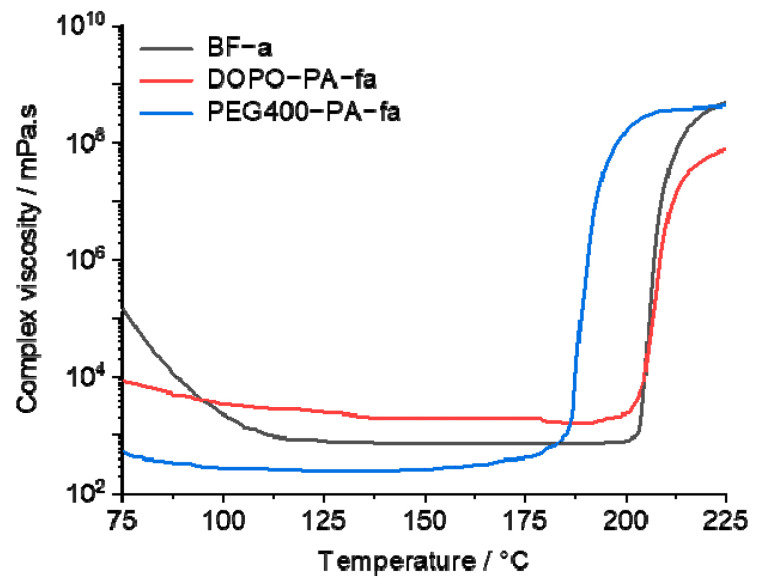
Evolution of the complex viscosity of each benzoxazine monomer as a function of the temperature.

Rheology was performed to investigate the polymerization progress (Figure 7). The recorded rheogram shows the evolution of the complex viscosity with increasing temperature. As mentioned before, PEG400-PA-fa starts to polymerize at a lower temperature as an increase in the complex viscosity was observed in the range of 175–185 °C, while DOPO-PA-fa and BF-a start to polymerize between 200–205 °C.

In summary, the benzoxazine monomer containing DOPO seems to delay the polymerization of the benzoxazine monomer. However, in spite of this delay, the range of the kinetics of polymerization is similar to traditional and commercial BF-a.

### 3.3. Thermo-Mechanical Properties of the Resulting Bisbenzoxazine Polymers

**Figure 8:**
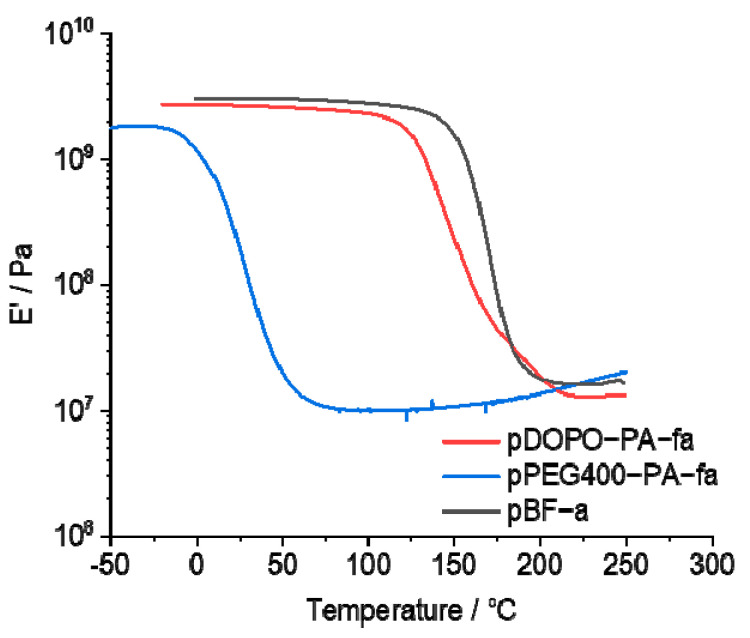
DMA results of pBF-a, pDOPO-PA-fa and pPEG400-PA-fa.

The glass transition temperature (*T*_g_) of the polymer pDOPO-PA-fa was measured at 150 °C (Figure S6 and Table 2), lower than the *T*_g_ of pBF-a (175 °C). It can be explained by the lower number of benzoxazine groups per grams of pDOPO-PA-fa compared with pBF-a. However, the rigid structure shows a beneficial effect on *T*_g_ when comparing with pPEG400-PA-fa (*T*_g_ = 26 °C). The storage modulus of pDOPO-PA-fa (2.7 GPa) at 20 °C is higher as for pPEG400-PA-fa (E_0_ = 1.2 GPa) as it already reached its *T*_g_ (Figure 8). While pDOPO-PA-fa and pPEG400-PA-fa contained a similar weight content of benzoxazine groups with 55% the bulkier DOPO-spacer group is improving the mechanical properties in comparison with the aliphatic PEG units. Thus, the DOPO linker increases significantly mechanical and thermal properties of the bio-based bisbenzoxazine. However, the storage modulus is slightly lower in comparison to pBF-a (3.0 GPa) with the highest benzoxazine proportion.

### 3.4. Thermal Stability and Thermal Degradation Kinetics

**Figure 9:**
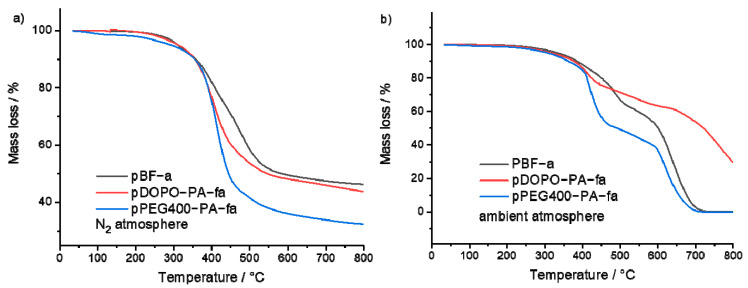
Thermogravimetric analysis of pBF-a, pDOPO-PA-fa and pPEG400-PA-fa under N_2_ (**a**) and ambient atmosphere (**b**).

The three polymers pBF-a, pDOPO-PA-fa and pPEG400-PA-fa all have a similar degradation profile under inert atmosphere (Figure 9a). The initial degradation temperature was 314 °C at a mass loss of 5 wt% (*T*_5%_) for pDOPO-PA-fa and pBF-a. pPEG400-PA-fa had a slightly reduced initial stability with *T*_5%_ = 291 °C. The evaluated temperature at 20 wt% mass loss showed that pDOPO-PA-fa and pPEG400-PA-fa show a comparable degradation temperature at 390 °C, whereas pBF-a is slightly more stable with *T*_20%_ = 400 °C. While pPEG400-PA-fa achieved a char yield of 32%, both pDOPO-PA-fa and pBF–a achieved a char yield of 44%. The incorporation of DOPO in the linker structure of the bisbenzoxazine has a beneficial effect on the thermal stability by significantly increasing the char yield in comparison with pPEG400-PA-fa.

In thermo-oxidative conditions, the polymers showed only slight differences for the initial degradation stability with 309 °C, 317 °C and 328 °C for pPEG400-PA-fa, pDOPO-PA-fa and pBF-a, respectively. However, at temperatures above 500 °C pDOPO-PA-fa was more stable and reached a char yield of 30% at 800 °C in contrast to no residual mass formation for pBF-a and pPEG400-PA-fa. This effect shows the advantage of the DOPO presence in the molecular structure as it shows a significant effect on the char yield formation at 800 °C under ambient atmosphere.

**Figure 10:**
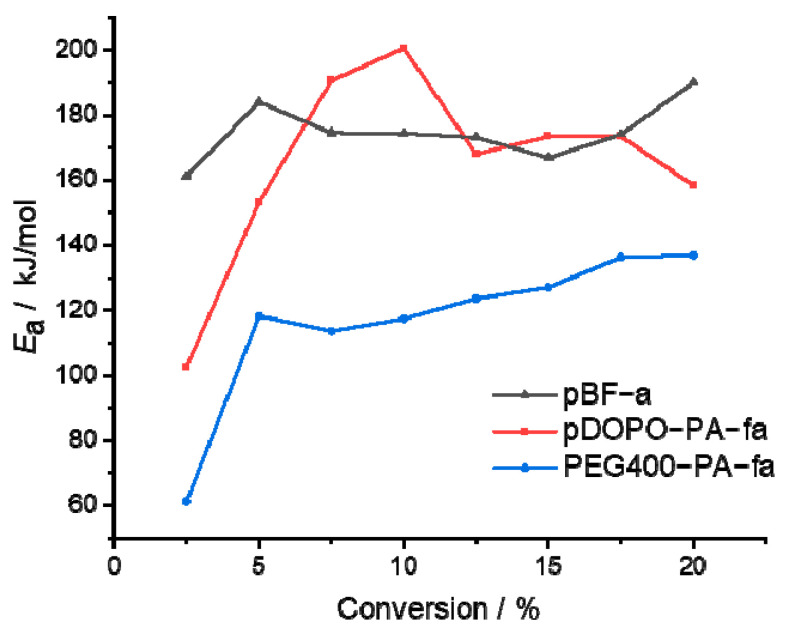
The degradation activation energy as a function of the degradation conversion of pDOPO-PA-fa, pBF-a and pPEG400-PA-fa, as determined by the Flynn–Wall–Ozawa equation.

Aside studying the degradation temperatures, the activation energy (*E*_a_) as an important kinetic parameter was derived from thermograms obtained under different heating regimes. *E*_a_ is directly related to the energetic needs for bond dissociation processes and indicates the tendency of material degradation during heating. The Flynn–Wall–Ozawa (FWO) method was used to calculate *E_a_* for pDOPO-PA-fa, pBF-a and pPEG400-PA-fa [36]. The FWO method is an isoconversional method that does not depend on the conversion function [37]. As long as the degradation mechanism stays the same for the investigated area, the method is not changing with different heating rates for different conversions. *E*_a_ (determined by −0.4567$\frac{E_{a}}{RT}$) can be obtained from the slope of $log_{10}\left(\beta \right)$ versus 1000/*T_c_* for different conversions obtained from TGA with different heating rates (Figure 10 and Figure S10). The FWO method can be expressed by the following Equation (1):

(1) $$log_{10}\left(\beta \right)=log_{10}\left(\frac{AE_{a}}{R\left(\alpha \right)}\right)-2.315-0.4567\frac{E_{a}}{RT_{c}}$$

where β is the heating rate, *T_c_* is the temperature at a conversion α, with *A* as frequency factor, *R* as gas constant and *E*_a_ as activation energy for the thermal degradation.

The study was performed by using different heating rates 2, 5, 10, 20 and 40 K/min for pDOPO-PA-fa, pBF-a and pPEG400-PA-fa (Figure S7). The investigated conversions (α) for each heating rate and polymer were 2.5, 5, 7.5, 10 12.5, 15, 17.5 and 20%.

For all studied compounds, a higher *E*_a_ was determined with increasing conversion. Thus, a higher energy is needed for further degradation. For a conversion of 20% *E*_a_ proved to be higher for the DOPO-containing pDOPO-PA-fa (*E*_a_ = 158 kJ/mol) in comparison to pPEG400-PA-fa (*E*_a_ = 136 kJ/mol). The reference pBF-a (*E*_a_ = 190 kJ/mol) needs more energy as it is slightly more stable than pDOPO-PA-fa. However, at a conversion of 10% the needed *E*_a_ for pDOPO-PA-fa (*E*_a_ = 200 kJ/mol) exceeds those of pBF-a (*E*_a_ = 172 kJ/mol).

### 3.5. Investigations on the Reaction against Small Flames

UL94 and LOI tests were conducted to investigate the reaction of each polymer against small flames (Table 3). According to the literature, pBF-a is achieving a V-1 rating with a total burning time of 24 s. pPEG400-PA-fa failed the test as it was burning longer than 60 s. This result is explained by the PEG backbone that widens the network and the less stable oxyethylene units. pDOPO-PA-fa achieved a V-0 rating with 1 s of total burning time, proving the efficiency of the incorporated DOPO spacer group.

As expected from the UL94 tests pPEG400-PA-fa achieved the lowest oxygen index (*OI*) with 21.5%. pBF-a achieved an *OI* of 26% and the pDOPO-PA-fa an *OI* of 35%. The significant improvement is according to the literature as a DOPO-, aniline- and phenol-based monomer with a phosphorous loading of 7.8 wt% achieved an *OI* of 40% [31].

Finally, the efficiency of DOPO-PA-fa to act as a flame retarding agent for other polymers was tested. For that purpose, it was blended with BF-a or PEG400-PA-fa (7 wt% of DOPO-PA-fa leading a phosphorous loading of 0.21 wt%) and compared to blends of BF-a and PEG400-PA-fa with the reactive additive DOPO-HQ resulting the same phosphorous loading of 0.21 wt%. Polymerized blends of DOPO-PA-fa and BF-a achieved an *OI* of 28%, while blends with PEG400-PA-fa an *OI* of 23.6%. Both *OI* values were increased by 2%, which is similar for the reactive additive as well (28.2% for pBF-a and 23.6% for pPEG400-PA-fa). This approach is promising as it shows a similar effectivity as the neat DOPO-HQ without being limited in regards to the loading.

## 4. Conclusions

This study describes the incorporation of a DOPO spacer group into bio-based benzoxazine monomer. DOPO-HQ was reacted with ethylene carbonate to promote its reactivity toward a solvent-free esterification with PA, to obtain a diphenol end-capped DOPO molecule. This precursor was reacted with furfurylamine and paraformaldehyde, leading to the benzoxazine monomer DOPO-PA-fa. The successful 4-step synthesis pathway was verified by NMR, IR and MS analysis.

The polymerization behavior and thermo-mechanical properties of the corresponding polymer were analyzed by DSC, DMA and rheological analysis and compared to BF-a and PEG400-PA-fa. Results showed that for DOPO-PA-fa, the polymerization starts at around 200 °C, alike to BF-a. Its *T*_g_ (150 °C) is lower than for rigid pBF-a (175 °C) but significantly higher than for the flexible pPEG400-PA-fa (20 °C).

The thermal stability and reaction tests against small flames showed the improved stability of pDOPO-PA-fa. While the thermal stability for the partly bio-based pDOPO-PA-fa under N_2_ atmosphere is already comparable to those of pBF-a, under ambient atmosphere it shows an increased stability with a char yield of 30% at 800 °C, whereas those for pBF-a and pPEG400-PA-fa are 0%. During UL94 test, pDOPO-PA-fa reached a V-0 rating and an OI of 35%. Thus, DOPO-PA-fa proved to be an interesting, partly bio-based benzoxazine monomer, with promising properties as an FR monomer for future applications.

## Figures and Tables

**Table 1:** DSC analysis of DOPO-PA-fa, BF-a and PEG400-PA-fa.

Formulation	*T*_onset_ [°C]	*T*_peak_ [°C]	∆*H* [J/g]	∆*H* [kJ/mol]
BF-a	213	236	293	127
DOPO-PA-fa	208	232	133	126
PEG400-PA-fa	177	217	118	112

**Table 2:** Dynamical mechanical properties of pBF-a, pDOPO-PA-fa and pPEG400-PA-fa.

Monomer	*E’*_20_ [GPa] ^a^	*E’*_100_ [GPa] ^b^	*T*_g_ [°C] ^c^
pBF-a	3.0	2.8	175
pDOPO-PA-fa	2.7	2.3	150
pPEG400-PA-fa	0.3	1.2 *	26

^a^ *E*’_20_ was determined at 20 °C; ^b^ *E*’ was determined at 100 °C; * measured at 0 °C; ^c^ *T*^g^ was determined by the maximum of *tan*(*δ*).

**Table 3:** Results of the UL94 and LOI burning tests for pBF-a, pDOPO-PA-fa and pPEG400-PA-fa.

Additive	Pwt%	Total Burning Time [s]	UL-94 Grade	LOI [%]
pBF-a	0	23.8 ± 4.4	V-1	26.1 ± 0.19
pDOPO-PA-faP	3.3	0.9 ± 0.5	V-0	35.0 ± 0.27
pPEG400-PA-fa		>180	n.b.	21.5 ± 0.11
pBF-a/DOPO-PA-fa/	0.21			27.9 ± 0.17
pBF-a/ DOPO-HQ	0.21			28.25 ± 0.15
pPEG400-PA-fa/DOPO-PA-fa/	0.21			23.6 ± 0.15
pPEG400-PA-fa/DOPO-HQ	0.21			23.6 ± 0.15

## Data Availability

Not applicable.

## Supplementary Materials

The following are available online at https://www.mdpi.com/article/10.3390/polym13244330/s1, Scheme S1: Synthesis of DOPO-OH, Figure S1: ^13^C NMR of DOPO-PA (DMSO-d_6_), Figure S2: ^31^P NMR of DOPO-PA, Figure S3: ^13^C NMR of DOPO-PA-fa (DMSO-d_6_), Figure S4: ^31^P NMR of DOPO-PA-fa, Figure S5: Thermogravimetric analysis of BF-a, DOPO-PA-fa and PEG400-PA-fa monomers under N_2_ (**a**) and ambient atmosphere (**b**), Figure S6: Tan(δ) results for pBF-a, pDOPO-PA-fa and pPEG400-PA-fa, Figure S7: TGA thermograms of pBF-a (**a**), pDOPO-PA-fa (**b**) and pPEG400-PA-fa (**c**) at different heating rates (2, 5, 10, 20 and 40 K/min), Figure S8: Flynn–Wall–Ozawa isoconversion plot for the calculation of activation energy for pBF-a (**a**), pDOPO-PA-fa (**b**) and pPEG400-PA-fa (**c**).
