# Supplementary material for: Bio-Based Bisbenzoxazines with Flame Retardant Linker
Source: Polymers (Basel). 2021 Dec 10;13(24):4330. doi: 10.3390/polym13244330 (PMC8707438; doi:10.3390/polym13244330)
Supplement: Supplementary file 1 [file polymers-13-04330-s001.zip › polymers-1432944-supplementary.pdf]

Biobased bisbenzoxazines with flame retardant linker

Thorben S. Haubold [1,2], Laura Puchot [3], Antoine Adjaoud [3,4], Pierre Verge [3] and Katharina Koschek[1]

Fraunhofer Institute for Manufacturing Technology and Advanced Materials IFAM, Wiener Strasse 12, 28359 Bremen, Germany; Thorben.haubold@ifam.fraunhofer.de (T.H.)

2 University of Bremen, Department 2 Biology/Chemistry, Leobener Straße 7, 28359 Bremen, Germany

3 Luxembourg Institute of Science and Technology, Materials Research and Technology Department, 5 Avenue des Hauts-Fourneaux, L-4362 Esch-sur-Alzette, Luxembourg; laura.puchot@list.lu (L.P.); Antoine.adjaoud@list.lu (A.A.); pierre.verge@list.lu (P.V.)

4 University of Luxembourg, 2, Avenue de l'Université, L-4365 Esch-sur-Alzette, Luxembourg

* Correspondence: Katharina.koschek@ifam.fraunhofer.de (K.K.)

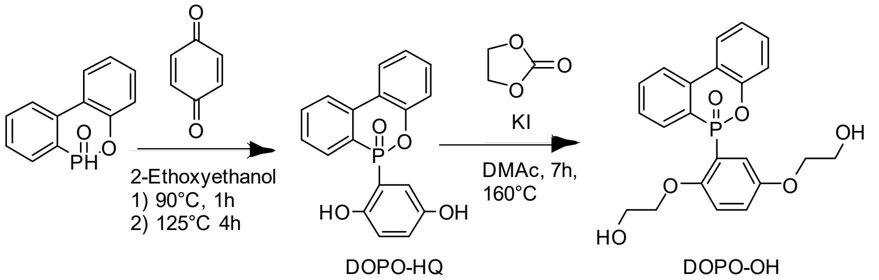

Scheme S1. Synthesis of DOPO-OH.

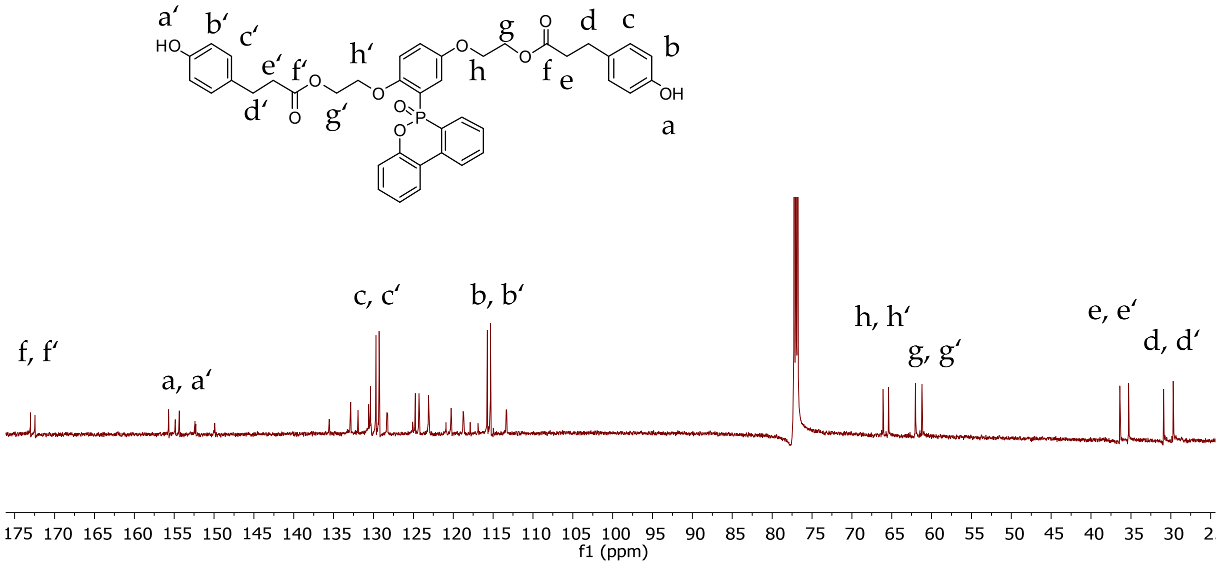

Figure S1. ^{13}C NMR of DOPO-PA (DMSO-d_6).

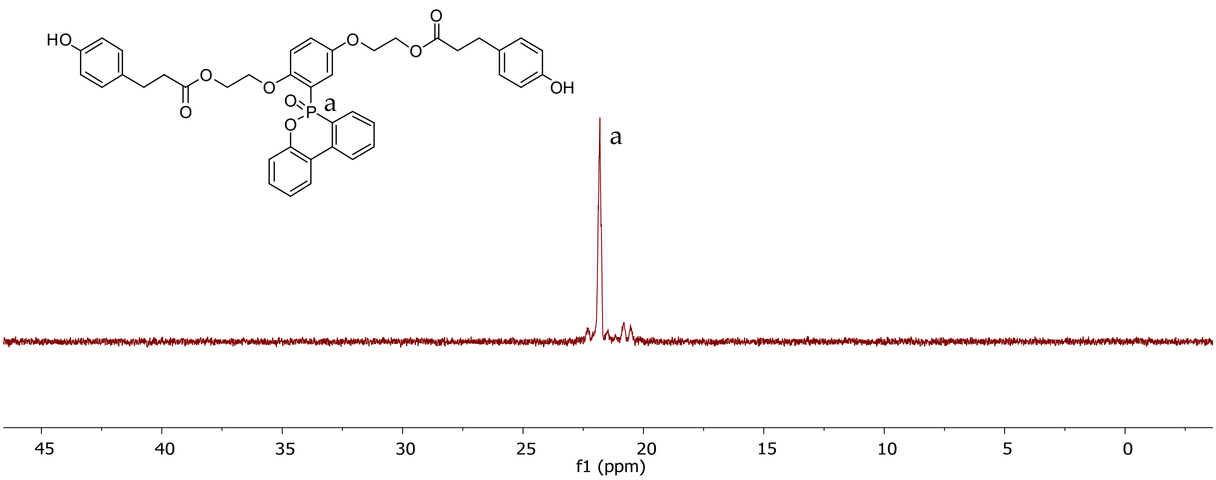

Figure S2. ^{31}P NMR of DOPO-PA.

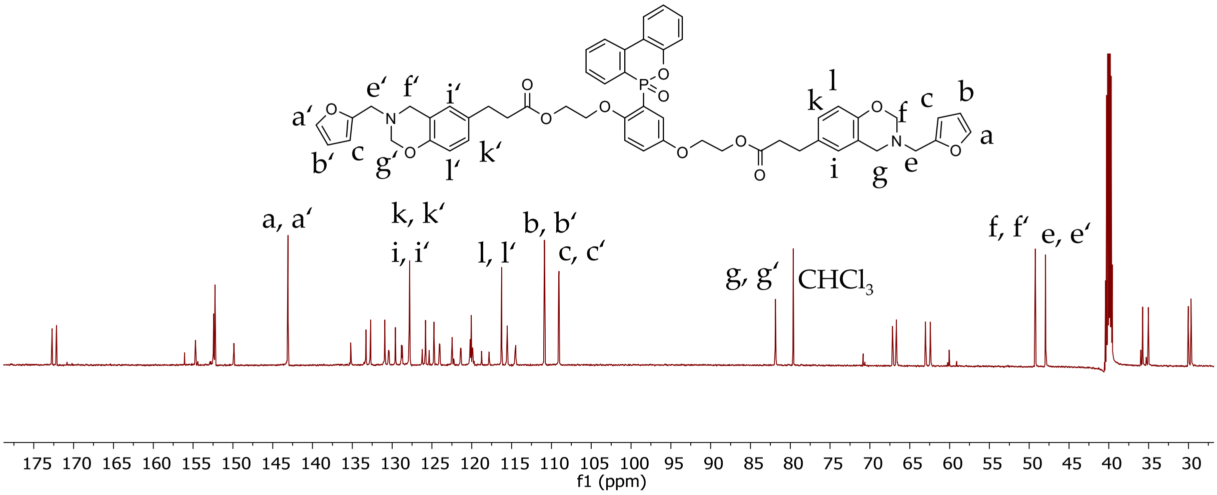

Figure S3. ^{13}C NMR of DOPO-PA-fa (DMSO-d_6).

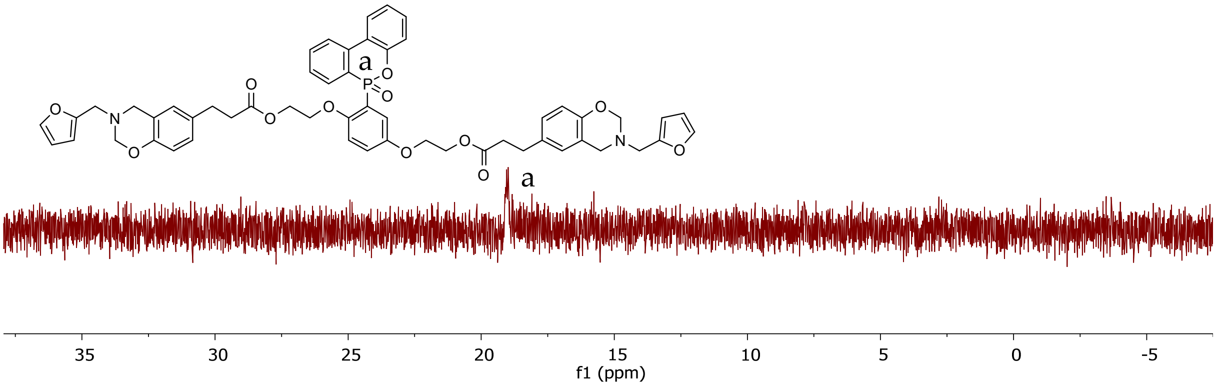

Figure S4. 31P NMR of DOPO-PA-fa.

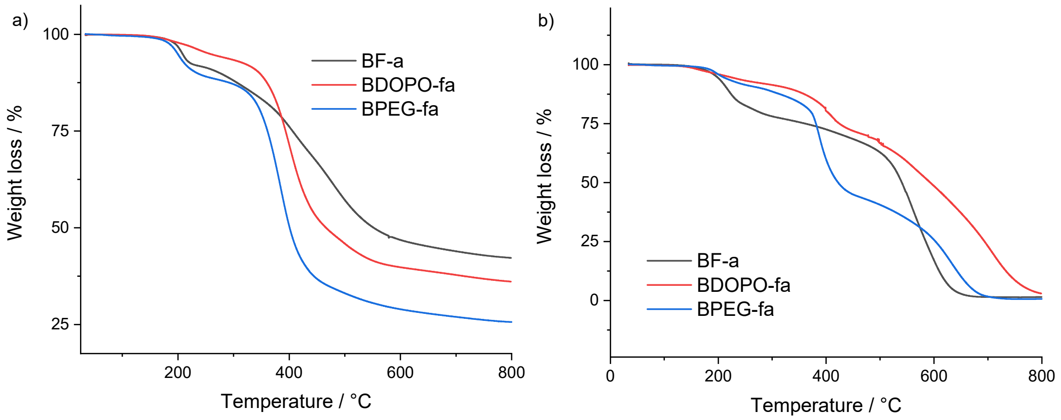

Figure S5. Thermogravimetric analysis of BF-a, DOPO-PA-fa and PEG400-PA-fa monomers under N2 (**a**) and ambient atmosphere (**b**).

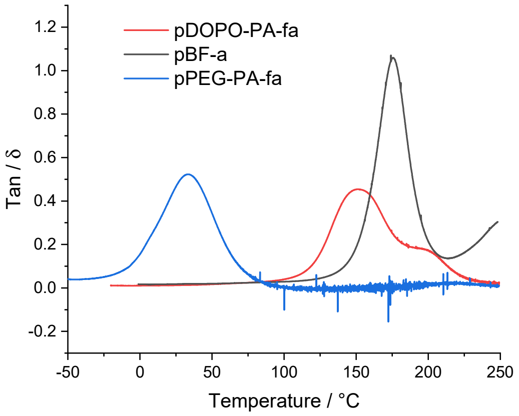

Figure S6. Tan(δ) results for pBF-a, pDOPO-PA-fa and pPEG400-PA-fa.

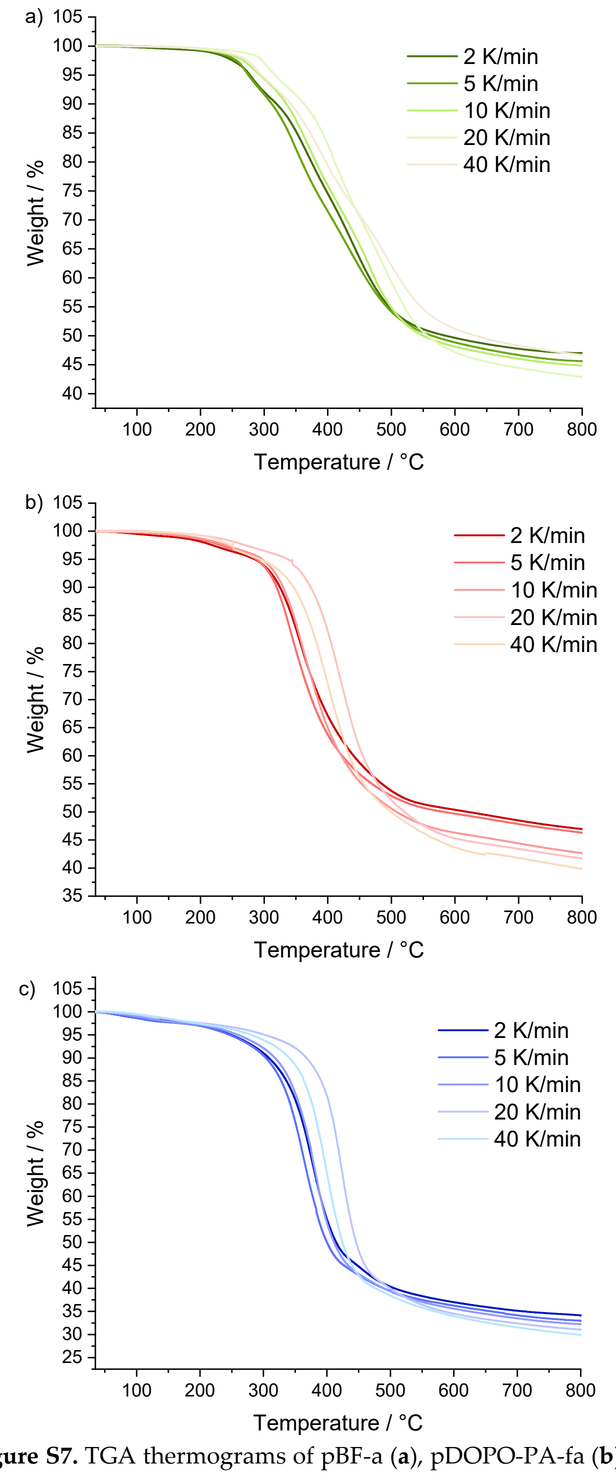

Figure S7. TGA thermograms of pBF-a (**a**), pDOPO-PA-fa (**b**) and pPEG400-PA-fa (**c**) at different heating rates (2, 5, 10, 20 and 40 K/min).

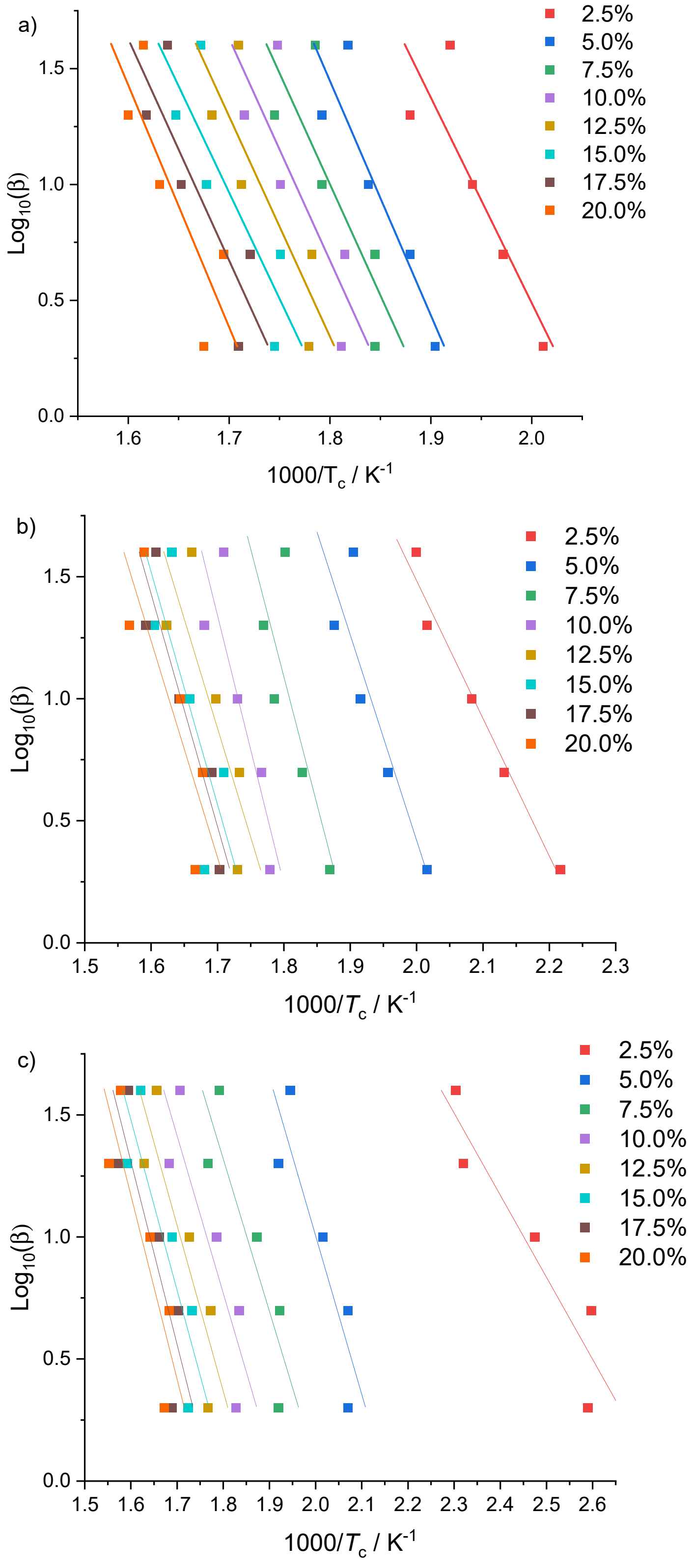

Figure S8. Flynn-Wall-Ozawa isoconversion plot for the calculation of activation energy for pBF-a (**a**), pDOPO-PA-fa (**b**) and pPEG400-PA-fa (**c**).
